# OnCampus: a mobile platform towards a smart campus

**DOI:** 10.1186/s40064-016-2608-4

**Published:** 2016-07-04

**Authors:** Xin Dong, Xiangjie Kong, Fulin Zhang, Zhen Chen, Jialiang Kang

**Affiliations:** 1 Chengdu College of University of Electronic Science and Technology of China, Chengdu, 611731 China; 2 School of Software, Dalian University of Technology, Dalian, 116620 China; 3 Library, Anshan Normal University, Anshan, 114007 China

**Keywords:** Smart city, Smart campus, Mobile application

## Abstract

An increasing number of researchers and practitioners are working to develop smart cities. Considerable attention has been paid to the college campus as it is an important component of smart cities. Consequently, the question of how to construct a smart campus has become a topical one. Here, we propose a scheme that can facilitate the construction of a smart and friendly campus. We primarily focus on three aspects of smart campuses. These are: the formation of social circles based on interests mining, the provision of educational guidance based on emotion analysis of information posted on a platform, and development of a secondary trading platform aimed at optimizing the allocation of campus resources. Based on these objectives, we designed and implemented a mobile platform called OnCampus as the first step towards the development of a smart campus that has been introduced in some colleges. We found that OnCampus could successfully accomplish the three above mentioned functions of a smart campus.

## Background

Information technology has brought tremendous changes to our lifestyles and living environments that are evident in daily life, and specifically, in areas such as communication, public security, energy, and water resources. Equipped with pervasive networks, advanced electronics, and various kinds of sensors, urban areas are evolving into smart cities. A smart city can be defined as “multiple sectors cooperating to achieve sustainable outcomes through the analysis of contextual real-time information shared among sector-specific information and operational technology systems” (Szabo et al. 2013). The findings of many studies have contributed towards the development of smart traffic systems, medical systems, and food systems that support the development of a smart city. As a special epitome of society, colleges constitute a significant element of cities.

Mobile devices evidently serve as ideal facilitating platforms for improving the efficiency of campus life (Chen et al. 2012). Because they are portable and easily accessible, they have become necessary devices used by millions of college students in their daily lives. Usage of mobile devices on campuses has become widespread in recent years. For instance, Mobile Virtual Campus (MVC) (Tan et al. 2010) and T3G (Chu et al. 2010) are two useful mobile learning tools available for college students. However, location-based service (LBS) is evidently more precise and reliable and has considerably improved the mobile living experience (Zhao et al. 2012). College students are perpetually fascinated by advanced technologies, especially smart phones. Studies have shown that the Internet is being accessed at rapid speeds with the increasing number of smart phones used within college campuses. Consequently, mobile platforms and LBS appear to be important considerations in developing smart campuses.

A college campus can be conceptualized as a scene comprising an area that is well-organized and contains high quality infrastructure. Additionally, because it is a long-term living space for students, it is a repository of students’ personal information and educational resources. Accordingly, the following characteristics of a smart campus are desirable. The first is accurate context awareness and ubiquitous access to network. The second is efficient resource allocation. The third characteristic is smart decision-making based on objective principles. Further, the ability of students to easily establish or enhance friendships based on intersecting circles is an important characteristic. Moreover, students’ freedom of speech should be safeguarded within a smart campus. However, a wide gap exists between this ideal vision and the current progress toward smart campus development. There have been many attempts to develop smart campuses that have focused on physical campus infrastructure and virtual intelligent services. Although these have considered many of the contextual aspects of campuses, most of their efforts have focused on the provision of physical equipment or functional applications. In our opinion, the development of parallel physical and virtual campus services is a less effective option than their interweaving in the construction of a smart campus. We have, therefore, proposed an innovative scheme to support the development of a smart campus based on certain theoretical concepts and academic approaches, such as interests mining, recommendation technology, and an educational guidance model. Based on this conceptual scheme, we designed and established an architecture for offering intelligent services. Our aim was to promote a smarter and friendlier campus as a living space compared with a conventional campus.

We have designed and developed a system named OnCampus that supports the creation of a smart campus through the use of mobile devices. The contributions of this initiative are threefold. First, we introduce a model for interests mining and establish interest-focused circles based on context awareness, location, students’ profiles, and relationships. Second, we present a platform that serves as a forum for users to share knowledge and post ideas. Managers can subsequently compile and analyze students’ posts on this platform. By exploiting such information, “campus emotion” can be analyzed and appropriate educational guidance can be provided. Third, we provide a solution for optimizing the usage of campus resources such as used books and surplus products.

The rest of this paper is organized as follows. The section on “Related work” introduces the literature on the current status of the research on smart campuses, as well as interests mining. The section on “Motivations and requirements” discusses our objectives relating to the design of a smart campus, and its requirements, including the provision of personalized services and scientific guidance, as well as optimization of resource use. In the next section, we discuss our proposed scheme that can contribute to the development of a smart campus. Based on this scheme, the following section discusses the design and implementation of a prototype that we developed for both the client interface (mobile phones) and the server interface (PCs). In the final section of the paper, we offer our conclusions.

## Related work

The idea of a smart campus is not a new one. However, more attention has focused on the infrastructure required for constructing smart campuses and related applications that involve both learning and teaching, as well as the provision of guidance on living to promote a satisfying personal experience of college life.

The design of smart campuses is commonly conceived from an educational and learning perspective. In 2010, a new conceptual paradigm pertaining to a novel holistic intelligent campus environment, termed iCampus (Ng et al. 2010) was proposed with the aim of enriching the end-to-end life cycle of learning within a knowledge ecosystem. Atif and Mathew (2013) have proposed a model of a smart campus that integrates real-world learning resources within a campus-wide social network. This model aims to embed learners within a smart campus environment that provides context-based personalized learning and feedback. Hirsch and Ng (2011) have proposed mobile cloud-based education as a method of ubiquitously providing contextually grounded learning using portable devices. Liu et al. (2014) also regard smart campus as an inevitable trend in the development of digital campuses. They have expounded a concept of smart campuses based on cloud computing and the internet of things, and have pointed out problems that can be observed after establishing application intelligence platforms.

A number of researchers have also focused on smart campus development based on data analysis. For example, Adamko and Kollar (2014) have conceived an intelligent campus that is based on data gathered from a crowd that are then analyzed, with subsequent provision of feedback as value-added services that satisfy users and in the meantime contributory as they would like to return. Boran et al. (2011) have described the implementation of a smart campus application prototype that integrates heterogeneous data using semantic technologies. Some studies have also focused on the social networking aspects of smart campus design. For example, Yu et al. (2011) have developed an architectural system based on service-oriented specifications to support social interactions within campus-wide environments. Based on their examination of the features and functions of WeChat, a social network platform, Xiang et al. (2015) have proposed a framework for university-based smart campus information dissemination.

Several of the existing smart campus initiatives^1^^,^ 2 have primarily been conceived from the perspective of high energy efficiency. Zhao et al. (2012) have proposed a resource-efficient, location-based mobile instant messaging system that can be applied in the context of a smart campus. Yim et al. (2014) have designed and implemented a video on demand system, entailing a smart campus guide as an android App that recognizes the structure that interests a user and displays useful information about that structure.

Although these projects are of practical value in their respective areas, their functionality is very limited based on either a drawing of a campus map or e-books. None of them facilitate college students through the depiction of the colorful campus life as a versatile, efficient, and convenient system.

Researchers focusing on interests mining have primarily engaged with topic models on web text such as blogs or microblogs. Very few studies have focused on interests mining conducted within campus environments. Kuang and Luo (2010) constructed a model for precisely and automatically identifying the interests of users from their logs and learning backgrounds, based on an e-learning system. Consequently, they were able to recommend resources related to these interests. Han and Xia (2014), who conducted a study on web log mining, proposed and demonstrated the efficiency and reliability of a data preprocessing method based on users’ characteristic interests. Further, Huayue (2012) applied a topic model to develop a model of users’ interests that enabled him to recommend resources for users of the system.

Although researchers are making concerted efforts to explore ways of building smart campuses, their development is still at a primary stage. Some researchers have analyzed characteristics and trends relating to smart campuses, noting the issues that need to be attended to in the process of constructing them. The key achievements to date are primarily in the areas of infrastructure development and service application. Others are attempting to improve the security or efficiency of campus life via cloud computing and the internet of things. These efforts, for example, in the area of navigation services, entail substantial benefits for users but are relatively restricted in their applications. Our initiative aims to contribute to the establishment of a smart campus and proposes an efficient scheme comprising interests mining, resource optimization and scientific guidance. In formulating this scheme, we propose a new concept, namely campus emotion, aimed at offering college students a better personal experience of campus life.

## Motivations and requirements

A college campus can be viewed as an environment that provides good infrastructure and conditions for providing services entailing contextual awareness. Moreover, it is a well-organized area in which a substantial number of users spend the majority of their time and generate a huge quantity of information or needs. This information is directly linked to the campus context, including its location and the profiles of its users. Desirable characteristics of a smart campus are accurate context awareness and ubiquitous access to networks, optimized and efficient use of a vast amount of resources, and smart predictions or decisions made on the basis of objective principles. We aim to offer college students a versatile and talented campus experience, while acquiring a practical grasp of campus life. Consequently, we have developed a feasible scheme to satisfy their requirements, thus enabling a smart campus to promote efficient and convenient college life for its users.

College students generally spend time communicating with their families, friends, and even strangers, to establish or enhance friendships. However, in most cases, students may not have a means to access what they want, or even to develop other interests. Interests mining, based on context awareness, locations, user profiles and proximity, and other relevant information can facilitate college students in satisfying their needs and making the most of available resources in school. In particular, faculty can make informed predictions and decisions when they can perceive a trend in the mood of the students, consequently providing them with more scientific and efficient guidance.

The following scenario illustrates the requirements that should be satisfied by a smart campus.

Bob was full of passion and curiosity when he entered university, dreaming about an enriching college experience. He found that students, researchers and many other agents performed various activities within the campus environment. Faced with an onslaught of information from multifarious social activities, he felt at sea as he did not know what really interested him. Propelled by his great passion, he chose to participate in some social activities and to join certain clubs because of the participation of his peers. However, he left these clubs because after some time he found that they did not match with his interests. The same mismatch occurred when he chose his profession 1 year later, but this time he could not leave. He no longer had the passion or desire to try new things. Moreover, he found it very difficult to conduct his research without a co-researcher or guide.

One day, he took part in a demonstration against the school, because he was invited by his roommate to do so and agreed with his complaints. When he graduated, he had to throw away some of the articles or books he had used on a regular basis which other students would have found useful and helpful.

Bob’s experience is the epitome of the typical college experience of most contemporary students. We surveyed students to learn about their daily lives to identify their needs. Our findings indicate that the needs of university students are diverse, encompassing their study, living and entertainment. Here, we present these needs in relation to the development of a smart campus.

1. *Personalized service* The most appropriate strategy in developing a smart campus is to tailor information and services to users’ interests and profiles. If this is not done, the information explosion may dampen the enthusiasm of campus users, including students and teachers. In the next section, we discuss a very effective proposed solution for offering personalized services based on interests mining.
2. *Scientific guidance* A community of college students, as a particular group of people, mostly comprises enthusiastic youth. Though they are well educated, the long duration of their school education has resulted in a lack of experience or good judgment. However, they are passionate and eager to make a contribution to their families and to society. All of these characteristics result in a strong tendency for them to become the cat’s paw. In the next section, we will introduce our vision of scientific guidance based on predictions of a community’s behavior and emotions. We also emphasize the role of what we term “campus emotion” in our scheme.
3. *Resource optimization* The concept of resources in a smart campus is not restricted to material resources. Information, contexts, events, and even users themselves, can serve as particular resources that should be optimized. Here, we verify a recommendation algorithm utilizing the user’s friendships as an effective strategy for optimizing resources.

## Proposed scheme

Our model entails the following two assumptions. The first is pervasive access to networks, whereby we assume that every user (e.g., students and faculty) is equipped with a personal device (a PC, smart mobile device, or PDA). Devices owned by users and the associated infrastructure form the basis of a smart campus system. In a campus situation, users maintain constant connections with each other, thereby benefiting from pervasive access to networks such as social and learning networks (Khabou et al. 2014). The second assumption relates to precise context awareness. In other words, we assume that a smart campus system has powerful capabilities regarding context awareness and pervasive computing (Khan and Zia 2007). Apart from users’ profiles, the environment users involved in such as building, location can be precisely obtained through advanced context extraction, identification, and integration for smart campus development.

Our proposed scheme entails interests mining, scientific guidance, and resource optimization based on the above assumptions.

As previously mentioned, we proposed incorporating interests mining into a smart campus to offer personalized services from a unique perspective. Differing from the state of art, interests mining is primarily based on a semantic analysis of web content or behavior. We consider the campus context, which includes a range of information relating to location, time, frequency, track, and accompany, to inferring users’ interests or needs. Subsequently, the formulation of a customized recommendation strategy enables the overload of information or resources to be filtered to provide a personalized service. For example, if a user frequently appears in the library and is always reading magazines about astronomy, users with similar interests can be recommended. This can be an effective method of finding potential friends or collaborators. From another perspective, this is also an example of resource optimization within a campus environment.

To provide students with scientific guidance, a smart campus should provide faculty with information on the students’ circumstances. A student community’s behavior and emotions can be predicted based on an analysis of the contexts of most students, combined with a technique for analyzing public opinion. We coined a new phrase, “campus emotion” to describe the evaluation of the emotional state of most students. If campus emotions are healthy or normal, this means that the mood of most students is positive. Conversely, if they show sickness, this reflects a negative mood among most students. For example, we can infer the impactive of some students through public opinion analysis. A customized scheme for providing guidance can be formulated by a supervisor.

We examined resource optimization from various aspects. Apart from mining potential resources for users, we are introducing a new recommendation algorithm to satisfy users’ needs. Based on a consideration of friendship, recommendations across different domains can be made and this work is being explored. This algorithm seems to perform better than the traditional algorithm.

To promote a smart campus, we propose a scheme comprising interests mining, scientific guidance, and resources optimization that corresponds to the above mentioned requirements. Based on our assumptions, the scheme entails a vision for the development of a smart campus. Moreover, we propose feasible possibilities, some of which are under investigation to verify whether they are efficient. Based on this scheme, we have designed and implemented a prototype that we have named “OnCampus”. This is discussed in detail in the following section.

## Prototype implementation

**Fig. 1:**
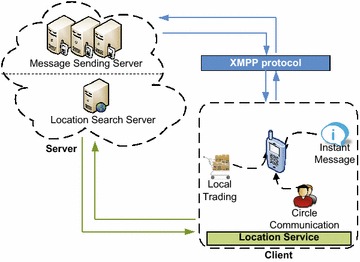
Architecture of OnCampus

We named the prototype OnCampus to proclaim our aim of offering college students a versatile and enriching campus experience. Figure 1 shows the architecture of OnCampus. This proposed architecture enables OnCampus to function as a practical and versatile campus assistant, archiving real-time messaging and data transmission. Over and above simple interactions between clients and servers, this architecture proposes location-based interactions as well as a rational communication mechanism. Servers constitute a two-tier structure for providing services and storing information. The first tier comprises a message-sending server, storing the information uploaded by mobile clients and transferring requested data to clients’ functional modules. The second tier is a location-searching server that merely collects information on locations and acquires clients’ location data by interacting with the clients’ location service modules. Clients include the interface of our OnCampus assistant that interacts with users. Three client modules cover important college activities. These are: circle communication, local trading, and a campus forum. With the introduction of LBS, OnCampus promotes the establishment and enhancement of friendships among students, knowledge exchange and affective interactions, and buying and selling used goods within a campus flea market.

### Communication mechanism

In the OnCampus, architecture, clients communicate with message-sending servers, exchanging service data, while they exchange location-based information with location-search servers (LSS). This communication frequently occurs via the XMPP protocol to archive real-time service and refresh data. Data responses obtained immediately after each refresh are not adequate in relation to energy consumption and network flow. Thus, we propose a practical communication mechanism to stipulate data transportation process between servers and clients. This section further illustrates the server structure and provides details of the communication process.

**Fig. 2:**
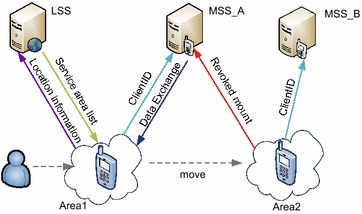
Process of communication between client and two-tier servers

To provide a complex LBS in an energy-saving and efficient way, OnCampus uses a two-tier server structure: a Location Search Server and a Message Sending Server to Client (LM2C), as shown in Fig. 2. This two-tier structure entails a message sending server (MSS) and a LSS. The general function of the server component of the system is to collect data, automatically classify and group information, and send messages to various locations. The LSS server specifically manages information on the physical area and identifies related MSS, whereas the function of the MSS is to store information and broadcast instant messages. Geographical areas such as Teaching Building 1, Teaching Building 2 and Stadium M, are partitioned by the LSS, and each physical area ID and its functional scope are documented as attributes of serving areas. Using the database of serving areas, it is easy to ascertain the assignment of a particular physical location to a particular serving area, thereby relating serving areas to a specified MSS. The MSS coordinates several areas, and maintains a dynamic user list according to users’ locations. Dealing with each physical area individually, the MSS is able to broadcast location-based instant messages.

Using the LM2C server, the OnCampus architecture regulates the process of data transfer between servers and clients to support system services. The communication process for establishing location-based instant messaging between clients and the two-tier servers is illustrated in Fig. 2. A client submits location information to LSS after connecting with it. LSS will then provide feedback on a set of data, including attributes of the surrounding area, MSS identifiers, as well as the corresponding relations of connection permission. After receiving the data, the client needs to determine the assigned MSS and wait to receive permission from the MSS to broadcast. The MSS adds the client to the broadcast list and then delivers permission-related information to the client. Messages will only be conveyed to clients who are included in the user list of a specified MSS. When a client on the user list of a particular MSS is out of the range of this MSS, the connection between the client and the MSS will be terminated and a connection request will be made to another MSS without communicating with the LSS.

This two-tier LM2C architecture is designed to accomplish energy-efficient conveyance of location-based instant messages. A set of areas will be designated to the client, as noted above. However, attributes associated with the exact locations of these areas will not be designated. This design feature enables an irredundant connection to be made to the LSS when the client’s location changes. The client can determine the next MSS according to given datasets. Thus, the OnCampus system entails an efficient method of using resources and server bandwidths. It further supports passive receipt of messages by clients, who are not required to initiate a process to obtain messages, thus reducing their energy consumption.

### OnCampus at a glance

Whether in terms of its architecture entailing the use of location contexts or its functional modules designed for clients, OnCampus offers a potentially feasible way to implement our above-described scheme. In this section, we discuss three functional modules aimed at service provision involving learning, living and entertainment. They are the “Group” module, the “Buy & Sell” module and the “Forum” module, all of which enable interests mining, scientific guidance, and resource optimization to be conducted. For example, data obtained from circle communication and local trading are conducive to mining users’ interests, enabling customized recommendations to be made. Moreover, inference of campus emotion within the forum enables the formulation of scientific guidance.

**Fig. 3:**
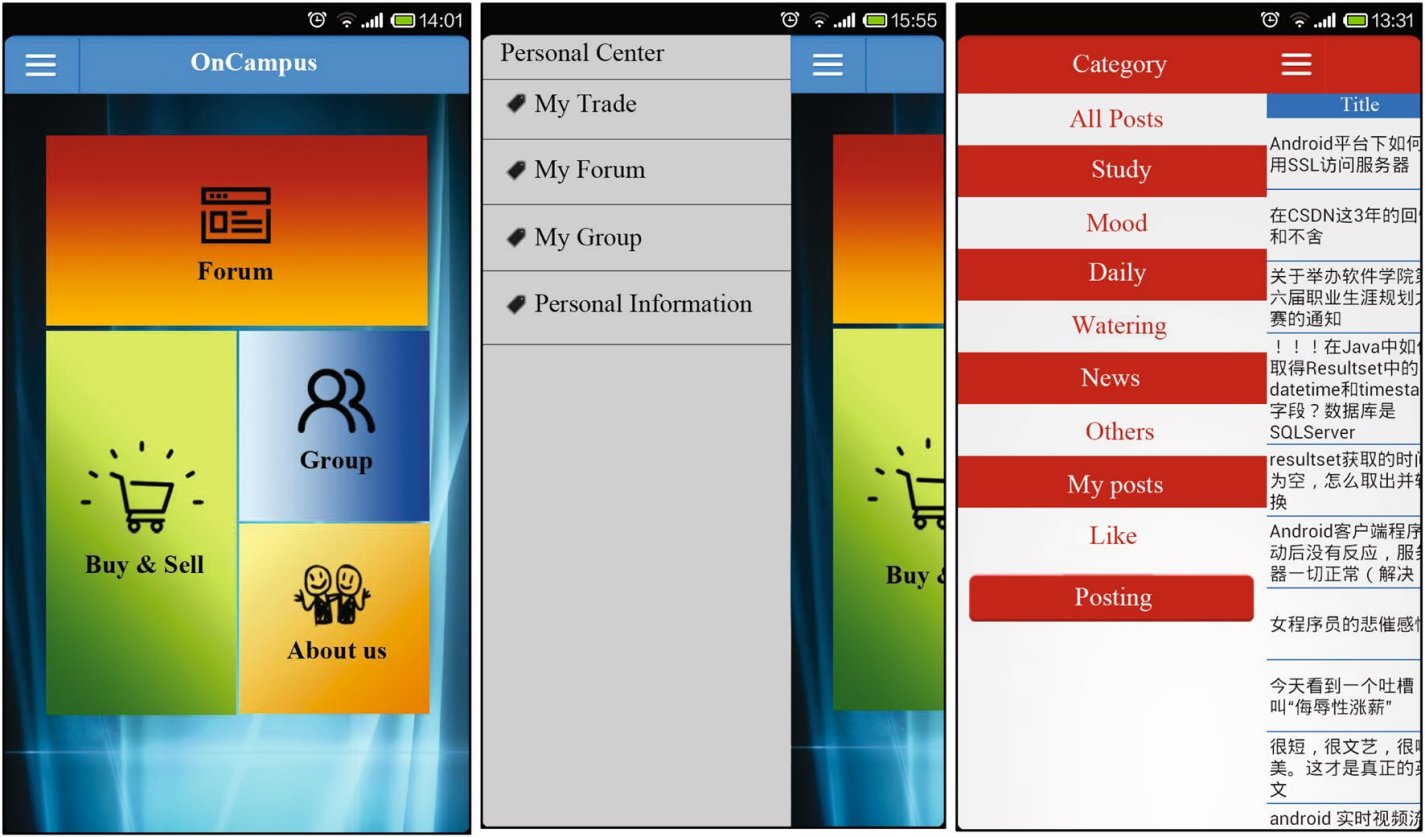
Implementation of the OnCampus prototype: main interface and “Forum”

**Fig. 4:**
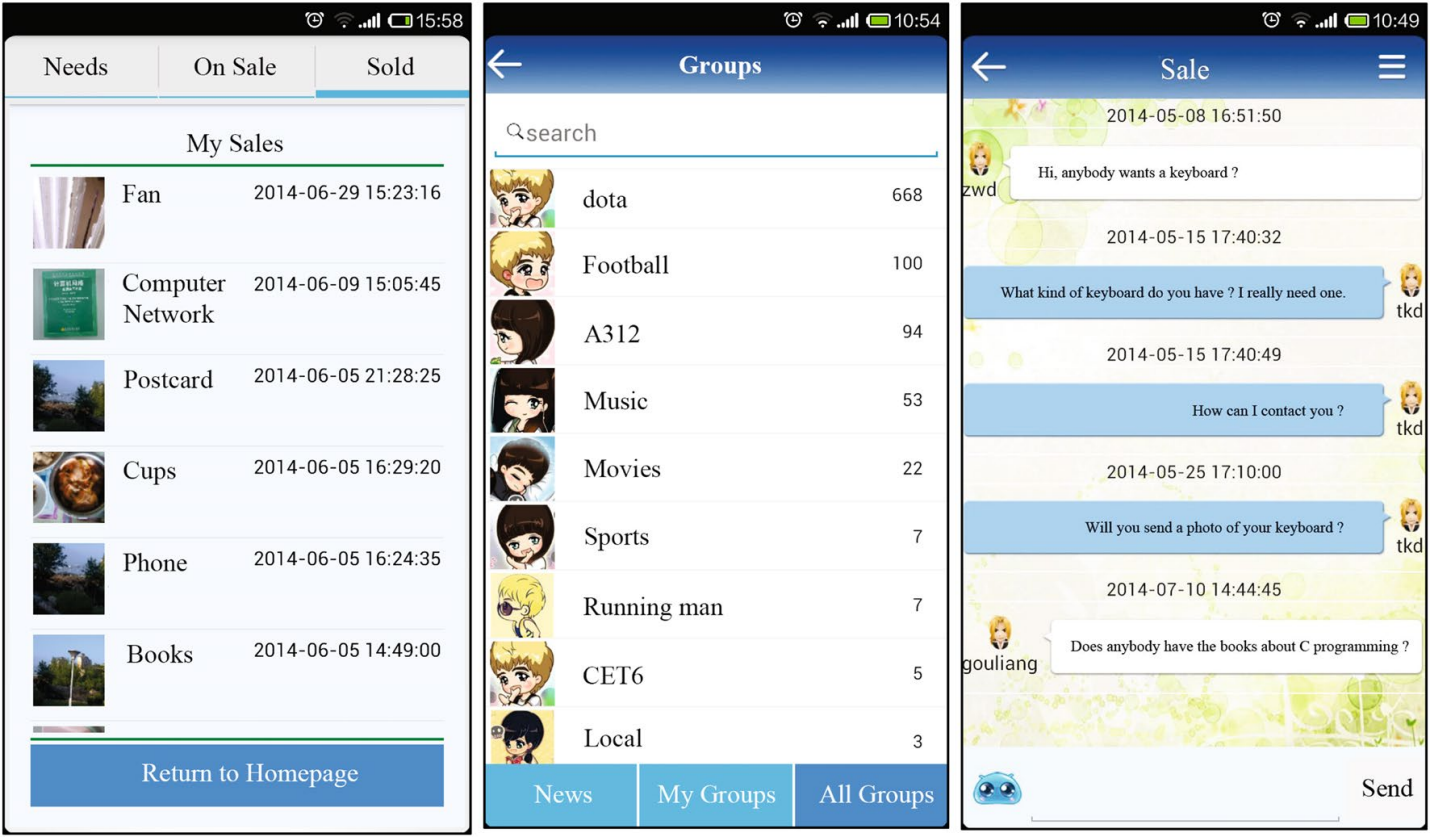
Implementation of the OnCampus prototype: “Group” and “Buy & Sell” modules

This OnCampus prototype was implemented on an android platform designed for the clients, while the server component was implemented using Java either on a Windows operating system or a Linux operating system. Figures 3 and 4 depict screenshots of part of the OnCampus interface.

The “Group” module provides highly popular social network functions within the campus, which meet the students’ needs relating to social communication. Circles with various topics can be developed for students to communicate with other users who have similar interests. This helps students to develop friendships, improve their perception, and broaden their horizons. As with some instant messaging systems, personal friends can be added, and status delivery, sharing, and retweeting within circles are also supported in this module. For example, users can show their current status as being happy, which can be viewed by everyone within their circles. Users’ friends have the option to reply, share, or forward this status.

The “Buy & Sell” module has a practical function within the campus, enabling students to exchange their goods. This module is designed to solve the problem of resource waste resulting from students discarding their commonly used articles or books that other students would find useful. Using the “Buy & Sell” module, students can broadcast information about what items they do not need and are willing to sell to others. Customers will thus be able to find what they need and complete transactions using the information provided in this module. Users have the ability to view product lists, search for certain products, release products offered for sale, and manage their lists of products for sale. Product lists are displayed according to various product categories. Consequently, students can scan a certain type of product using easy slide on type tabs. The products in these lists are usually designated as being for sale, whereas products that have already been sold can either be marked accordingly or deleted from the lists as desired by owners.

The “Forum” module is a practical application for knowledge sharing, affective interaction and developing “Q & A” sessions. This module is designed to provide a platform for students to share their knowledge, broadcast news, express their feelings, and ask for help. Any registered OnCampus member can read and reply to the posts. The posters can delete, edit, or share their posts. They have full privileges to manage them. Everyone here enjoys complete freedom of speech.

The design and implementation of OnCampus is of great significance. OnCampus offers optimal convenience and efficiency to college students. The practicability of this initiative is demonstrated by its wide range of useful applications within campus life. For example, Jack maybe very interested in skiing and is looking for students with similar interests. One solution is to look for student circles that have discussed this topic in OnCampus’s “Group” module. If Jack cannot find any such circles, he can himself develop a circle focusing on the topic of skiing to attract students. He will develop strong friendships who share his hobbies within this circle. Another example can be cited involving a new computer keyboard. Students would rather sell their old but functional keyboards using the “Buy & Sell” module to other students who need this item in an economical and environmental friendly way. There are many scenarios within universities in which convenience is valued, and OnCampus can offer this convenience in such circumstances.

### Statistics and an analysis of the OnCampus application

OnCampus can provide college students with intelligent assistance, not only because of its practical client-server architecture, but also because of its energy-efficient characteristics. The use of the LM2C server structure to provide LBS can save energy. Specifically, when first connecting to LSS, a client receives extra information for computing MSS without the need to repeatedly connect to this LSS. This communication mechanism is designed to save energy. An experiment involving five repetitions was conducted to evaluate its performance on three different smart phones with the same configurations. Phone 1 was set to run no applications to obtain a standard standby time. Phone 2 used our OnCampus prototype to calculate location and server-based information, and Phone 3 used a controlled prototype without the LM2C architecture that communicated each time with the server to identify its location. The results of the experiment revealed that on three occasions, our architecture performed better than the prototype without the LM2C architecture. The experimental settings and results have been published (Zhao et al. 2012).

**Fig. 5:**
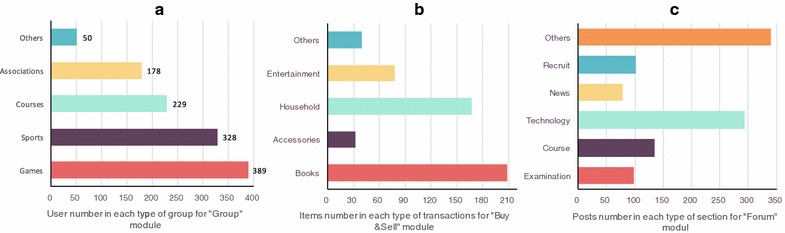
Statistical data on the usage of each module

OnCampus has been distributed and applied over the last 6 months within the School of Software at the Dalian University of Technology. We conducted a survey on the uses of each module. There were 2102 registered users in total, of whom 1166 users (about 55.47 %) were active. The statistical data obtained for each module are shown in Fig. 5. For the “Group” module, there were 67 student-created groups. We divided these into five categories: games (e.g., “LOL” and “DOTA”), sports (e.g., football and badminton), courses (e.g., software engineering, C++ programming), associations (e.g., drama clubs and dance groups) and others. Figure 5a indicates that a large number of students participated in the games group. It seems that the registered users preferred campus games to sports and courses. In addition, student associations did not attract as many members as we had anticipated. Thus, in preliminary ideas, more school activities should be encouraged. Moreover, our results showed that there were 526 items posted in relation to the OnCampus “Buy & Sell” module, as shown in Fig. 5b. Most of these items were books and household products. Figure 5c shows the number of posts for each category of the “Forum” module. Evidently, discussions relating to the topic of professional technology accounted for a significant proportion of all the discussions. The content and topic distribution may to some extent reflect the students’ psychology from a theoretical perspective.

Apart from the substantial services offered by OnCampus, and its efficiency, it has considerable potential for actualizing our scheme toward the development of a smart campus. Specifically, users’ interests can be identified through the use of the OnCampus system, especially within the “Group” module. Data and information are generated when users interact with other circle members. Information is available on location, user track, proximity, activities, and other relevant topics. Moreover, data on preferences and profile data can be collected when users use the “Buy & Sell” and “Forum” modules. All of these kinds of information have unpredictable potential in relation to the implementation of the scheme that we have presented here.

## Conclusion

In this paper, we have suggested that smart campuses are an inevitable trend in the context of smart cities. We have proposed a feasible scheme entailing interests mining, scientific guidance, and optimization of resource use within an initiative that contributes toward the development of smart campuses. We designed and implemented a prototype system based on our proposed OnCampus scheme. We adopted the XMPP protocol and our proposed LM2C structure to implement the system in a manner that was energy saving and efficient. We further developed three functional modules, namely “Group”, “Buy & Sell”, and “Forum” modules to provide services related to learning, living, and entertainment. OnCampus can significantly contribute in the following ways: through the creation of social circles based on interests mining, the provision of educational guidance based on emotion analysis, as an information-sharing platform, and by developing a secondary trading platform aimed at the optimal allocation of campus resources.

The application of OnCampus enables the conduct of interests mining, the provision of scientific guidance, and the optimization of resources. For instance, data from communication occurring within student circles and local trading are conducive to mining users’ interests, thus enabling the customization of recommendations. Moreover, scientific guidance can be formulated if we can infer campus emotions reflected on the campus forum. Future work will engage with these issues.

## Footnotes

1
http://greensmartcampus.eu/.

2
http://smartgridcenter.tamu.edu/seci/.
