# Erratum to: OnCampus: a mobile platform towards a smart campus

**DOI:** 10.1186/s40064-016-3388-6

**Published:** 2016-10-11

**Authors:** Xin Dong, Xiangjie Kong, Fuli Zhang, Zhen Chen, Jialiang Kang

**Affiliations:** 1 Chengdu College of University of Electronic Science and Technology of China, Chengdu, 611731 China; 2 School of Software, Dalian University of Technology, Dalian, 116620 China; 3 Library, Anshan Normal University, Anshan, 114007 China

## Erratum to: SpringerPlus (2016) 5:974 DOI 10.1186/s40064-016-2608-4

Upon publication, it was noticed that in the original version of the article (Dong et al. 2016), Fuli Zhang’s name was incorrectly given as Fulin Zhang. This has now been corrected in this erratum.
